# Increased Expression of NXPH4 Correlates with Immune Cell Infiltration and Unfavorable Prognosis in Hepatocellular Carcinoma

**DOI:** 10.1155/2022/5005747

**Published:** 2022-10-06

**Authors:** Qin Tang, Yue-ming Chen, Mei-mei Shen, Wen Dai, Hang Liang, Jun-nan Liu, Jian Gao

**Affiliations:** ^1^Department of Gastroenterology, Second Affiliated Hospital, Chongqing Medical University, Chongqing, China; ^2^Key Laboratory of Diagnosis and Treatment of Severe Hepato-Pancreatic Diseases of Zhejiang Province, The First Affiliated Hospital of Wenzhou Medical University, Zhejiang, China; ^3^Bashu Secondary School, Chongqing, China; ^4^Chongqing Medical University, Chongqing, China

## Abstract

Hepatocellular carcinoma (HCC) is one of the leading malignant carcinomas. Despite the advancement in the treatment for HCC, such as precise hepatectomy, radiotherapy, transarterial therapies, chemotherapy, targeted treatments, and immunotherapy, the 5-year overall survival rate of HCC is extremely low. Hence, novel biomarkers are urgently needed for advancing the therapy and prognosis of HCC. Neurexophilin 4 (NXPH4) is a neuropeptide-like glycoprotein. The study is designed to investigate the function of NXPH4 in HCC through a comprehensive bioinformatics analysis. NXPH4 expression status and prognostic values were analyzed via multiple datasets, such as TCGA, GEO, and ICGC. The association between NXPH4 and immune cell infiltration was estimated by TIMER, TISIDB, and CIBERSORT. In vitro, we explored the biological function of NXPH4 in JHH7 and SNU182 cells through knocking down the expression of NXPH4 via siRNA. In general, NXPH4 was predominantly upregulated in HCC tumors, and increased NXPH4 expression predicted unfavorable prognosis. The gene enrichment analysis displayed that NXPH4 was related with metabolic pathways. NXPH4 expression was correlated with immune cell infiltration. NXPH4 knockdown significantly suppressed proliferation, migration, and invasion of JHH7 and SNU182 cells. This study suggested that the upregulation of NXPH4 is associated with adverse prognosis and immune cell infiltration in HCC. NXPH4 could be a novel biomarker of unfavorable prognosis and an underlying target for immunotherapy in HCC.

## 1. Introduction

Hepatocellular carcinoma (HCC) is one of the malignant cancers with high morbidity and mortality. According to global statistics, the number of newly diagnosed liver cancer patients and liver cancer-related deaths worldwide was 905,677 and 830,180, respectively, in 2020 [1, 2]. Primary liver cancer mainly includes HCC, intrahepatic cholangiocarcinoma, and mixed hepatocellular carcinoma, of which HCC accounts for more than 75% to 85% [3]. HCC pathogenesis is correlated with certain pivotal risk factors, such as hepatitis virus infection, alcohol abuse, and metabolic abnormalities. Despite the advancement in the treatment for HCC, including precise hepatectomy, radiotherapy, transarterial therapies, chemotherapy, targeted treatments, and immunotherapy, the 5-year overall survival rate of HCC is extremely low [4]. The uncertainty of the molecular mechanisms and lack of disease specific markers have dramatically hindered the diagnosis and therapy of HCC [5]. Therefore, novel biomarkers are urgently needed for advancing the prognosis and therapy of HCC.

Neurexophilin (NXPH) family is comprised of NXPH1/2/3/4 members [6]. As endogenous ligands of *α*-neurexin in the brain, NXPH is an important regulator of neuronal cells and participates in intracellular signaling transduction [7–9]. NXPH1 has been confirmed as an underlying biomarker for invasion and metastasis in breast cancer [10]. A previous research presented that NXPH2 expression in macrophages was upregulated during immune system exhaustion [11]. The members of NXPH family has been reported as unfavorable prognosis biomarkers in cancers, including papillary glioneuronal tumor, leukemia, pancreatic ductal adenocarcinoma, breast cancer, and bladder cancer [12–16]. Neurexophilin 4 (NXPH4) is a neuropeptide-like glycoprotein and is widely expressed among human organs, including the brain and liver. NXPH4 is expressed in neuronal cells and responsible for eating, mood, balance, and movement. NXPH4 has a similar domain structure of NXPH1 and interacts with *α*-neurexin [17]. As a ligand for neurexin, it has the ability to regulate synaptic inhibitory neurotransmission in cerebellar Golgi granule cells [8]. Currently, the role of NXPH4 in cancer has attracted attention of some researchers. NXPH4 knockdown significantly suppressed cell proliferation and migration of non-small-cell lung cancer [18]. Recently, there was a study revealed that NXPH4 is an underlying indicator for early diagnosis of HCC [19].

Currently, the function of NXPH4 in HCC is not available. Therefore, we conducted an integrated bioinformatic analysis for NXPH4 expression in HCC in multiple databases, including The Cancer Genome Atlas (TCGA), International Cancer Genome Consortium (ICGC), Gene Expression Omnibus (GEO), and Tumor Immunity Estimation Resource (TIMER). We estimated the prognostic value of NXPH4 using Kaplan-Meier plotter database. To further understand the underlying pathogenic mechanism of NXPH4 in HCC, we explored NXPH4 coexpression genes via GO and KEGG pathways. Additionally, the association between NXPH4 expression and immune cell infiltration was estimated. Lastly, we investigated biological function of NXPH4 in JHH7 and SNU182 cells through knocking down the expression of NXPH4 via siRNA. Our study demonstrates that NXPH4 plays an essential effect on immune cell infiltration and prognosis of HCC. Therefore, we speculate that NXPH4 could be a promising biomarker for unfavorable prognosis and an underlying target for immunotherapy in HCC.

## 2. Materials and Methods

### 2.1. Gene Expression and Prognostic Analysis

Firstly, we estimated the expression levels of NXPH4 in 33 cancers in TCGA dataset by TIMER database (http://timer.cistrome.org). Next, we downloaded the transcriptional expression and clinic data from the TCGA-LIHC. The RNA-Seq data included 371 tumor specimens and 50 adjacent normal specimens; 50 of them were matched specimens. We also chose two different databases to sever as the validation cohorts. The GSE64041 dataset was downloaded from GEO, which included 60 HCC samples, 5 healthy samples, and 60 adjacent normal samples; 60 of them were matched samples. The ICGC-LIRI-JP dataset was obtained from the ICGC Data Portal, comprising 206 HCC samples and 177 adjacent normal samples; 175 of them were matched samples. All data were integrated via R 4.1.3. We performed the assessment of NXPH4 expression in HCC and normal samples. We compared the NXPH4 expression between different subgroups. These figures were visualized by the R package “ggpubr” and “ggplot2.” We estimated the expression level of NXPH4 on TP53 mutation status and methylation status using UALCAN database (http://ualcan. path. uab.edu/). We assessed the relationship between NXPH4 expression and survival in HCC via Kaplan-Meier plotter database (https://kmplot.com/analysis/).

### 2.2. Coexpressed Genes and Regulatory Networks of NXPH4

We selected the top five coexpressed genes of NXPH4 using cBioPortal database (https://www.cbioportal.org/). The GEPIA2 database (http://gepia2.cancer-pku.cn) was utilized to perform the survival analysis of the top five genes in HCC. According to GO and KEGG enrichment analyses, we evaluated the functions and pathways associated with the top 200 coexpression genes of NXPH4 via DAVID database (https://david.ncifcrf.gov).

### 2.3. Immune Cell Infiltration Analysis

Based on the TCGA-LICH cohort, we utilized CIBERSORT to calculate the relative fraction of tumor infiltrating immune cells (TIICs). HCC specimens were grouped into high- and low-NXPH4 groups depended on median expression value of NXPH4. The relative abundance of TIICs was compared in different NXPH4 expression groups. We utilized TIMER database (http://timer.cistrome.org) to perform the correlation analysis between NXPH4 expression and various immune cells and their gene markers. We used TISIDB (http://cis.hku.hk/) to evaluate the association of NXPH4 expression levels and immune inhibitors, chemokine, and chemokine receptors in HCC.

### 2.4. Cell Culture and Transfection

We purchased human HCC cell lines JHH7 and SNU182 from Meisen CTCC and Qida (Shanghai, China), respectively. Cells were cultured in DMEM (JHH7) with 10% fetal bovine serum (FBS, Gibco) or RPMI-1640 (SNU182) with 10% FBS (Qida, Shanghai) at 37°C in a 5% CO_2_ atmosphere. Both cell lines were certified by short tandem repeat (STR) analysis (Meisen CTCC, Zhejiang, China). Before transfection, cells were cultured to approximately 50% confluence in 6-well plate. We used Lipofectamine 2000 transfection reagent (Grand Island, NY, USA) to transfect the small interfering RNA (siRNA) (GenePharma, Shanghai, China). After transfection with siRNA for 48 hours, RT-PCR was performed to examine the transfection efficiency. The sequences of siRNA were as follows: siRNA1: 5′-UCUGUAUCUUCGUCUCUUUTT-3′ (forward) and 5′-AAAGAGACGAAGAUACAGAUG-3′ (reverse), siRNA2: 5′-UAAGACUGUAAAGGCCUAATT-3′ (forward) and 5′-UUAGGCCUUUACAGUCUUAGG-3′ (reverse), and negative control (NC): 5′-UUCUCCGAACGUGUCACGUTT-3′ (forward) and 5′-ACGUGACACGUUCGGAGAATT-3′ (reverse).

### 2.5. RNA Extraction and Real-time Quantitative Polymerase Chain Reaction

TRIzol was purchased from Invitrogen (Grand Island, NY, USA) for the extraction of total RNA. The HiScript III RT SuperMix for qPCR Kit (Vazyme) was used to perform the reverse transcription. Thunderbird SYBR qPCR Mix (Vazyme) was utilized to analyze real-time quantitative polymerase chain reaction (RT-qPCR). The *β*-actin was employed to control relative expression of NXPH4. The primer sequences were as follows: NXPH4: 5′-TGCCAAGCCCTTCAAAGTCATCTG-3′ (forward) and 5′-GTGCTCACTCTGGAAGTTATAGTCTGG-3′ (reverse) and *β*-actin: 5′-CTCTTCCAGCCTTCCTTCCT-3′ (forward) and 5′-AGCACTGTGTTGGCGTACAG-3′ (reverse). The fold change in RNA expression was measured by the 2 − *ΔΔ*Ct method.

### 2.6. Cell Proliferation Assay

Cell Counting Kit-8 (APExBIO, USA) assay: after interfered with siRNA for 24 h, transfected cells were plated in 96-well plates at a density of 2 × 10^3^ cells/well. From the first to the fifth day, the serum-free medium of 10% CCK-8 reaction solution was used to replace the original medium and incubated for 1 h. The light absorbance was quantified at 450 nm (OD-450).

### 2.7. Cell Migration and Invasion Assays

Chambers and Matrigel were purchased from BD Biosciences and Corning (NY, USA), respectively. Briefly, 6 × 10^4^ cells were plated in the upper chambers with Matrigel coated to estimate tumor invasion, and the chambers without Matrigel were used to assess tumor cell migration. In a 24-well plate, the upper wells were added with 200 *μ*L serum-free medium, and the lower wells were added with 800 *μ*L medium containing 10% FBS. The cells were incubated for 24-48 hours. At the observation time point, the cells were cleared from the surface of the upper chambers' membrane with a cotton swab. The invasive/migratory cells were fixed using methanol and stained by 0.1% crystal violet. The quantity of cells was calculated in 5 different areas under a microscope.

### 2.8. Statistical Analysis

R version 4.1.3 and GraphPad Prism v9.0.2 (GraphPad software, LLC, USA) were used for all statistical analyses. Student's *t*-test was employed to estimate two group comparisons of continuous variables. Nonparametric test was analyzed by Kruskal-Wallis test and Wilcox test. Univariate and multivariate Cox analyses were utilized to screen potential prognostic factors. The *p* < 0.05 was considered statistically significant.

## 3. Results

### 3.1. Transcriptional Level of NXPH4

**Figure 1:**
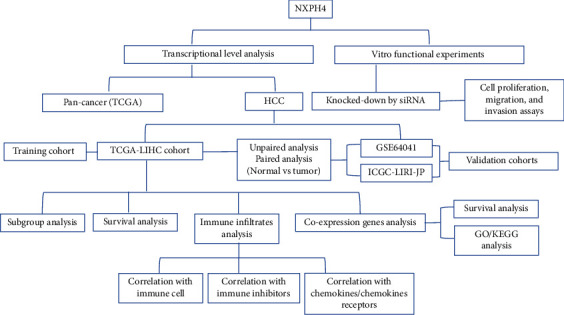
Flow diagram of the study.

**Figure 2:**
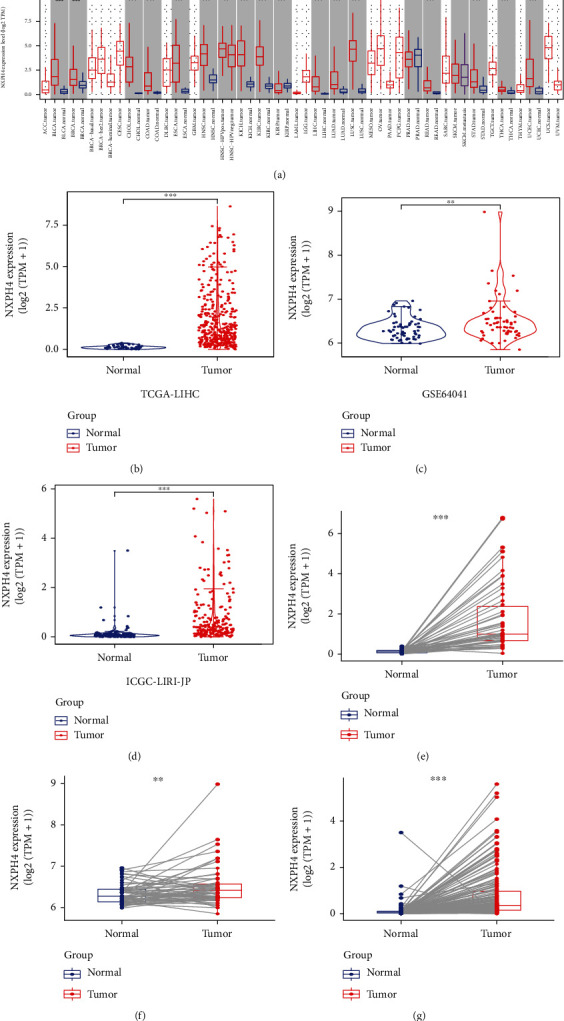
(a) The expression levels of NXPH4 across TCGA cancers (with tumor and normal). (b–d) Based on different datasets, unpaired analyses showed the mRNA expression of NXPH4 in HCC and normal (adjacent) samples: (b) TCGA-LIHC; (c) GSE64041; (d) ICGC-LIRI-JP. (e–g) Paired analysis showed the mRNA expression levels NXPH4 in HCC and normal (adjacent) samples: (e) TCGA-LIHC (*n* = 50); (f) GSE64041 (*n* = 60); (g) ICGC-LIRI-JP (*n* = 175). ^∗∗^*p* < 0.01 and ^∗∗∗^*p* < 0.001.

This study was performed based on the flow diagram in Figure 1. We estimated the expression status of NXPH4 in pan-cancers using TIMER database **(**Figure 2(a)**)**. The results showed that compared with normal samples, the NXPH4 expression was obviously higher in tumor samples, including HCC, bladder cancer, colon cancer, breast cancer, and gastric cancer. However, a significantly low expression was found in kidney renal papillary cell carcinoma (KIRP).

Subsequently, we evaluated the mRNA expression of NXPH4 on TCGA-LIHC cohort. The result revealed that the NXPH4 expression was apparently higher in HCC specimens (*n* = 371) as compared to normal specimens (*n* = 50) (Figure 2(b), *p* < 0.001). To further clarify the result, we chose another two independent external datasets, including GSE64041 dataset and ICGC-LIRI-JP dataset as validation cohorts to analyze NXPH4 expression levels in HCC (Figures 2(c) and 2(d)). The results demonstrated that NXPH4 expression was remarkably higher in HCC specimens than normal specimens (*p* < 0.005). The increased mRNA level of NXPH4 in HCC was also confirmed in paired data analysis (Figures 2(e)–2(g)). Overall, the outcomes of several datasets revealed that the NXPH4 expression in HCC samples was considerably higher than in normal samples.

### 3.2. Correlation between the NXPH4 Expression and Clinicopathological Characteristics

**Figure 3:**
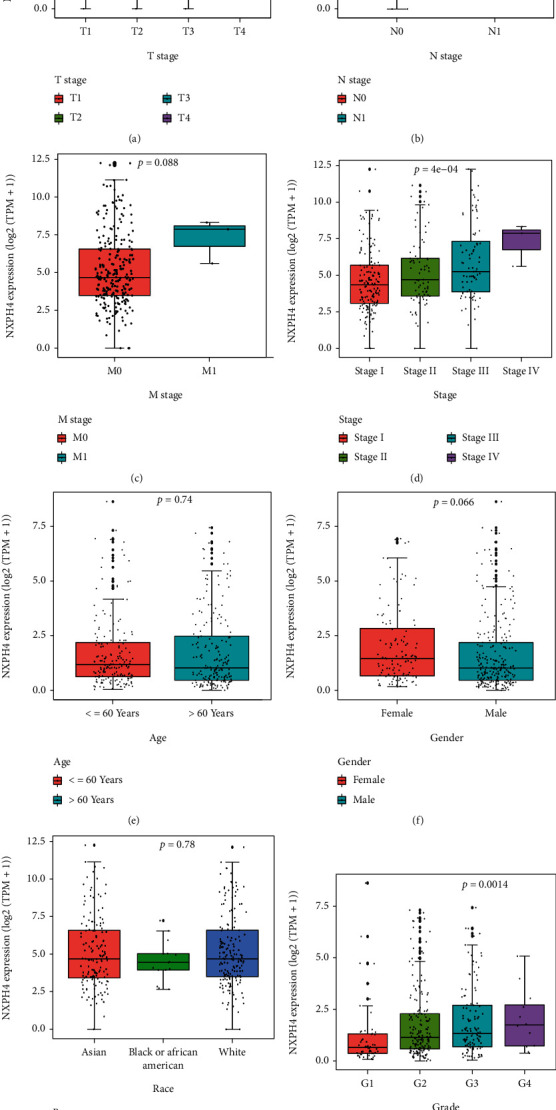
(a–h) The boxplot showed the correlation between the relative NXPH4 expression and clinicopathological parameters: (a) T stage (Kruskal-Wallis test); (b) N stage (Wilcox test); (c) M stage (Wilcox test); (d) stage (Kruskal-Wallis Test); (e) Age (Wilcox test); (f) gender (Wilcox test); (g) race (Wilcox test); (h) grade (Kruskal-Wallis test).

We estimated the correlation between NXPH4 expression and clinicopathological features in HCC patients. The findings showed that increased expression of NXPH4 was related with T stage (Figure 3(a), *p* = 0.00051), N stage (Figure 3(b), *p* = 0.045), clinical stage (Figure 3(d), *p* = 4*e* − 04), and grade (Figure 3(h), *p* = 0.0014). But the correlation between the expression of NXPH4 and M stage is not significant (Figure 3(c), *p* = 0.088). The reason might be that the common metastasis of HCC is intrahepatic metastasis rather than distant metastasis, as well as few M1 samples (*n* = 3). There is no significant correlation with age, gender, and race groups (Figures 3(e)–3(g)). Interestingly, we found a high level of NXPH4 in TP53-mutated HCC compared with TP53-mild type (Supplementary: Figure S1A, *p* = 0.049). Taken together, these data declared that NXPH4 might be a crucial factor in promoting the malignancy of HCC. To further explore the aberrant upregulated expression of NXPH4 in HCC, we estimated the methylation status of NXPH4 in HCC through UALCAN databases. The result displayed that the methylation level of NXPH4 was lower in tumor samples as compared to normal samples, but the difference was not significant (Supplementary: Figure S1B, *p* = 0.145).

### 3.3. Increased NXPH4 Expression Relates with Unfavorable Prognosis in HCC Patients

**Figure 4:**
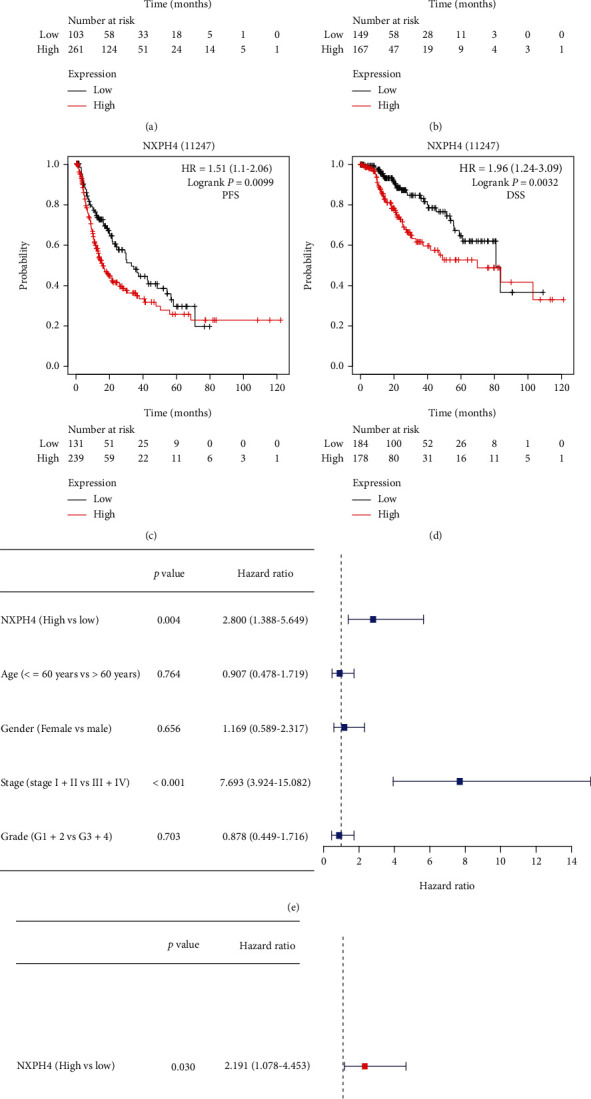
(a–d) The prognostic value of NXPH4 in HCC (Kaplan-Meier plotter): (a) over survival (OR); (b) recurrence free survival (RFS); (c) progression free survival (PFS); (d) disease specific survival (DSS); (e) univariate analysis of NXPH4 expression and clinical parameters; (f) multivariate analysis of NXPH4 expression and clinical parameters.

We assessed the prognostic value of NXPH4 in HCC with Kaplan-Meier plotter. The findings demonstrated that increased NXPH4 expression was strongly associated with adverse survival in HCC, including overall survival (OS, Figure 4(a), *p* = 0.00052), recurrence free survival (RFS, Figure 4(b), *p* = 0.044), progression free survival (PFS, Figure 4(c), *p* = 0.0099), and disease specific survival (DSS, Figure 4(d), *p* = 0.0032). Furthermore, univariate analysis discovered that increased NXPH4 expression was clearly related with unfavorable OS (HR = 2.8, 95% CI = 1.388–5.649, *p* = 0.004, Figure 4(e)). The conclusion was further confirmed by multivariate Cox regression analyses (Figure 4(f)). These results suggested that NXPH4 was an independent unfavorable prognostic factor in patients with HCC.

### 3.4. GO and KEGG Enrichment Analyses of Coexpressed Genes

**Figure 5:**
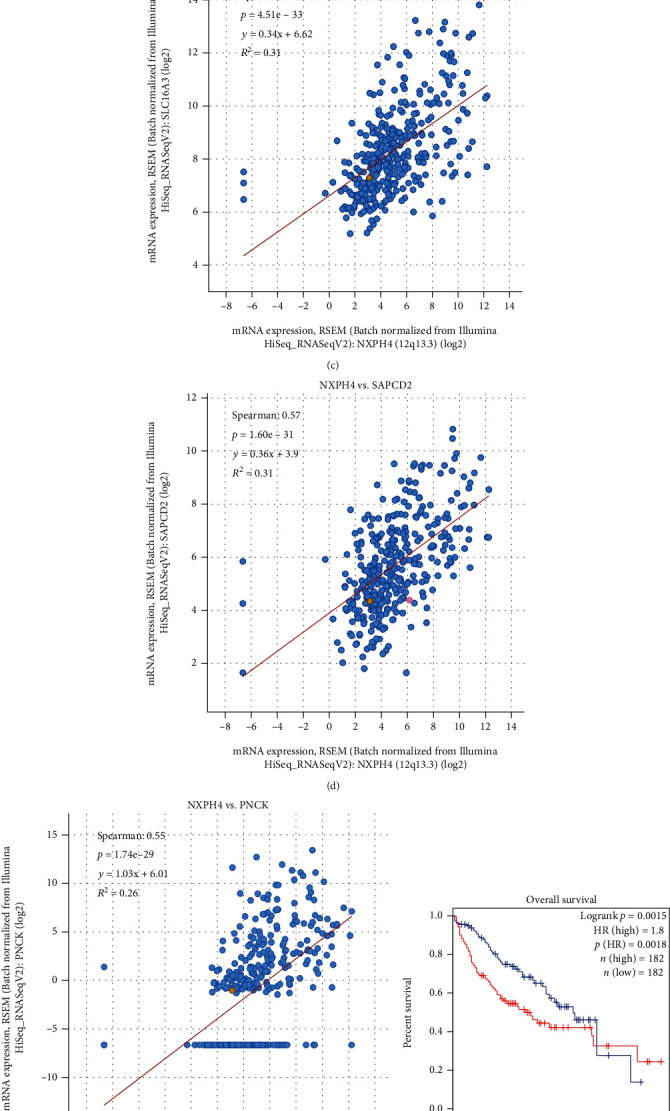
(a–e) The correlation analysis of the top 5 coexpressed genes of NXPH4 (cBioPortal): (a) PKM; (b) ENO2; (c) SLC16A3; (d) SAPCD2; (e) PNCK. (f–j) The over survival analysis of the top 5 genes in HCC (GEPIA2): (f) PKM; (g) ENO2; (h) SLC16A3; (i) SAPCD2; (j) PNCK.

In general, coexpressed genes have similar effects. We utilized the cBioPortal database to screen the NXPH4 coexpressed genes in the TCGA-LIHC cohort. The top five coexpressed genes of NXPH4 were selected by Spearman's correlation with an adjusted *p* value. The top five genes positively correlated with NXPH4 were pyruvate kinase (PKM, *r* = 0.62, *p* = 6.12*e* − 38), enolase 2 (ENO2, *r* = 0.59, *p* = 1.86*e* − 33), solute carrier family 16 member 3 (SLC16A3, *r* = 0.58, *p* = 4.51*e* − 33), suppressor APC domain containing 2 (SAPCD2, *r* = 0.57, *p* = 1.60*e* − 31), and pregnancy upregulated nonubiquitous CaM kinase (PNCK, *r* = 0.55, *p* = 1.74*e* − 29) (Figures 5(a)–5(e)). The overall survival analysis of top five coexpression genes showed that increased expression of these genes was correlated with unfavorable prognosis of HCC (Figures 5(f)–5(j)).

To identify the underlying pathogenic mechanism of NXPH4 in HCC, we analyzed the top 200 coexpressed genes of NXPH4 via GO and KEGG pathway analyses. The key biological processes (BP) were nervous system development, cell migration, brain development, and regulation of cell proliferation (Supplementary: FigureS2A). The critical cellular components (CC) were plasma membrane, cytosol, membrane, and integral component of plasma membrane (Supplementary: FigureS2B). The genes coexpressed with NXPH4 were predominant in regulating molecular functions (MF) of protein binding, cadherin binding, calmodulin binding, and kinase activity (Supplementary: FigureS2C). As for KEGG pathways, the main enrichment pathway was “metabolic pathways” (Supplementary: FigureS2D). Overall, the results suggested that the NXPH4 coexpressed genes were majorly related to intracellular signaling transduction and metabolic processes that are crucial in HCC development.

### 3.5. Relationship between NXPH4 Expression and Immune Cell Infiltration in HCC

**Figure 6:**
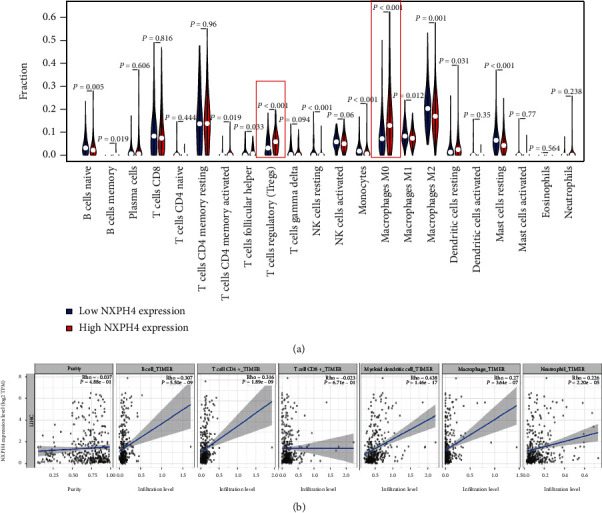
(a) The violin graph shows the difference of immune cell in the high and low NXPH4 expression groups. (b) The correlation between NXPH4 expression and the degree of immune cell infiltration.

Since NXPH4 is upregulated in HCC and closely related with unfavorable prognosis, NXPH4 might play a prooncogenic role in HCC. It was reported that the member of NXPH family affects the immune system [11, 16]. We investigated whether NXPH4 plays a pathogenic role in the immunity of HCC. Firstly, we assessed the distribution of 22 TIICs in different NXPH4 expression groups (Figure 6(a)). The findings revealed that compared with high NXPH4 expression patients, the infiltration levels of B cells naïve, monocytes, resting mast cells, NK cells, M1 macrophages, and M2 macrophages were obviously higher in patients with low NXPH4 expression. On the contrary, the infiltration of regulatory T cells (Tregs), M0 macrophages, and resting dendritic cells was significantly lower in HCC with low NXPH4 expression than in patients with high NXPH4 expression.

Then, we used TIMER to estimate the association between NXPH4 expression and infiltrating immune cells in HCC (Figure 6(b)). NXPH4 expression was positively related with the infiltrating levels of B cells (*r* = 0.307, *p* = 5.50*e* − 09), macrophages (*r* = 0.27, *p* = 3.64*e* − 7), neutrophils (*r* = 0.226, *p* = 2.20*e* − 05), CD4 + T cells (*r* = 0.316, *p* = 1.89*e* − 09), and dendritic cells (DCs, *r* = 0.438, *p* = 1.46*e* − 17) in HCC tissues, while NXPH4 expression appeared to have no apparent relationship with CD8+ T cells and tumor purity (*p* > 0.05). Like NXPH4, the top 3 positive correlation genes (PKM, ENO2, and SLC16A3) were strongly associated with B cell, macrophages, CD4 + T cells, DCs, and neutrophils of HCC samples (*p* < 0.05) (Supplementary: Figure S3). Furthermore, we also used TIMER to investigate the underlying association between NXPH4 expression and TIIC gene markers in HCC (Supplementary: TableS1). Table S1 showed that NXPH4 was obviously correlated with these gene markers, such as CD4+ T cell, T cell (general), monocytes, neutrophils, Tregs, and T cell exhaustion. Notably, programmed death-1 (PD-1), cytotoxic T-lymphocyte antigen-4 (CTLA-4), mucin domain-containing protein-3 (TIM-3), and lymphocyte activation gene-3 (LAG3) are the key genes participating in T cell exhaustion and have essential effects on antitumor immunotherapies. This research declared that NXPH4 was positively related with four genes, including TIM-3 (*r* = 0.4, *p* < 0.001), PD-1 (*r* = 0.288, *p* < 0.001), CTLA4 (*r* = 0.285, *p* < 0.001), and LAG3 (*r* = 0.155, *p* < 0.01). These results demonstrated that NXPH4 affects the development and progression of HCC through regulating immune cell infiltration and may be utilized as a novel immunotherapy target.

**Figure 7:**
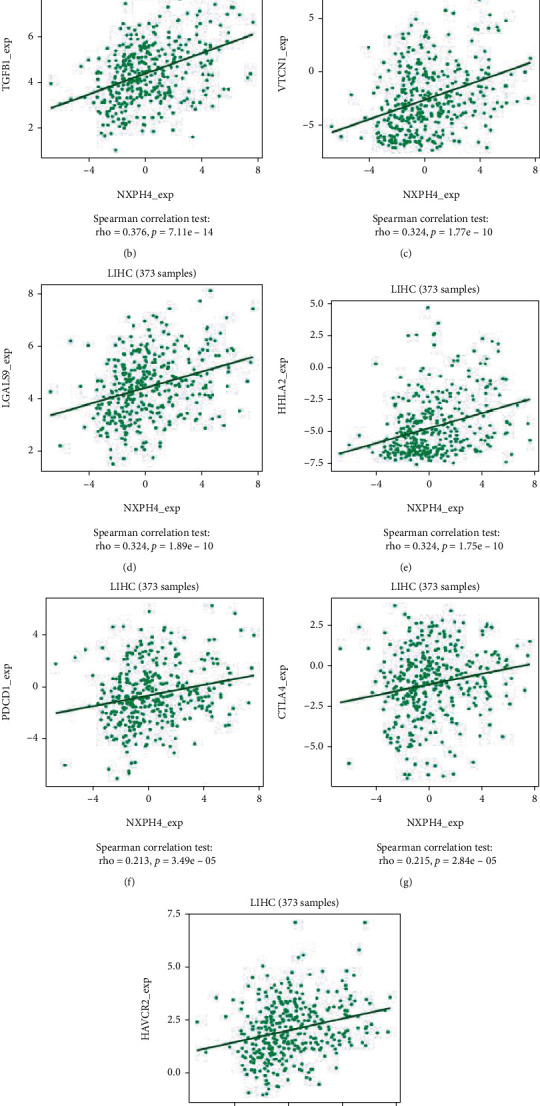
Correlation analysis between NXPH4 expression and immune inhibitors. (a) Heat map analysis of the correlation between NXPH4 and immune inhibitors in tumors. (b–h) Immune inhibitors: NXPH4 expression in HCC is positively correlated with TGFB1, VTCN1, LGALS9, HHLA2, PDCD1, CTLA4, and HAVCR2. Blue and red color stand for negative and positive correlations, separately. (i) Differences in the proportions of 7 immune checkpoints in the HCC specimens in the high and low NXPH4 expression groups.

In tumor microenvironment (TME), increased expression of inhibitory immune checkpoints and elevated immunosuppressive cytokines correlated with developing and maintaining immunosuppressive microenvironment and promoted evasion of tumoral cells [20]. Consequently, we assessed the correlation between NXPH4 expression and immune inhibitors in HCC through TISIDB database. The heat maps displayed the association between NXPH4 expression and immune inhibitors in cancers (Figure 7(a)). The results revealed that NXPH4 expression has positive correlations with TGFB1 (rho = 0.376, *p* = 7.11*e* − 14), VTCN1 (rho = 0.324, *p* = 1.77*e* − 10), LGALS9 (rho = 0.324, *p* = 1.89*e* − 10), HHLA2 (rho = 0.324, *p* = 1.75*e* − 10), PDCD1 (rho = 0.213, *p* = 3.49*e* − 05), CTLA4 (rho = 0.215, *p* = 2.84*e* − 05), and TIM3 (HAVCR2) (rho = 0.278, *p* = 5.22*e* − 08) in HCC (Figures 7(b)–7(h)), whereas NXPH4 expression was not correlated with LAG3, programmed cell death protein 1 ligand 2(PDCD1LG2), and T cell immunoreceptor with immunoglobulin and ITIM domains (TIGIT). It is well known that PD-1 (PDCD1), CTLA4, TIM3 (HAVCR2), LAG3, PDL1 (CD274), PDCD1LG2, and TIGIT are important inhibitory immune checkpoints related to immune escape. Therefore, we further used TCGA-LIHC cohort to assess the expression of these immune checkpoints in different NXPH4 expression level groups **(**Figure 7(i)). The results indicated that compared with low NXPH4 expression patients, the expression levels of PDCD1, TIGIT, CTLA4, HAVCR2, and LAG3 were markedly higher in patients with high NXPH4 expression. Nevertheless, the expression of CD274 and PDCD1LG2 was e no remarkable difference in both groups. These results demonstrated that NXPH4 may regulate immune inhibitors to inhibit the immune response in HCC, thereby impacting the immune cell infiltration in TME.

**Figure 8:**
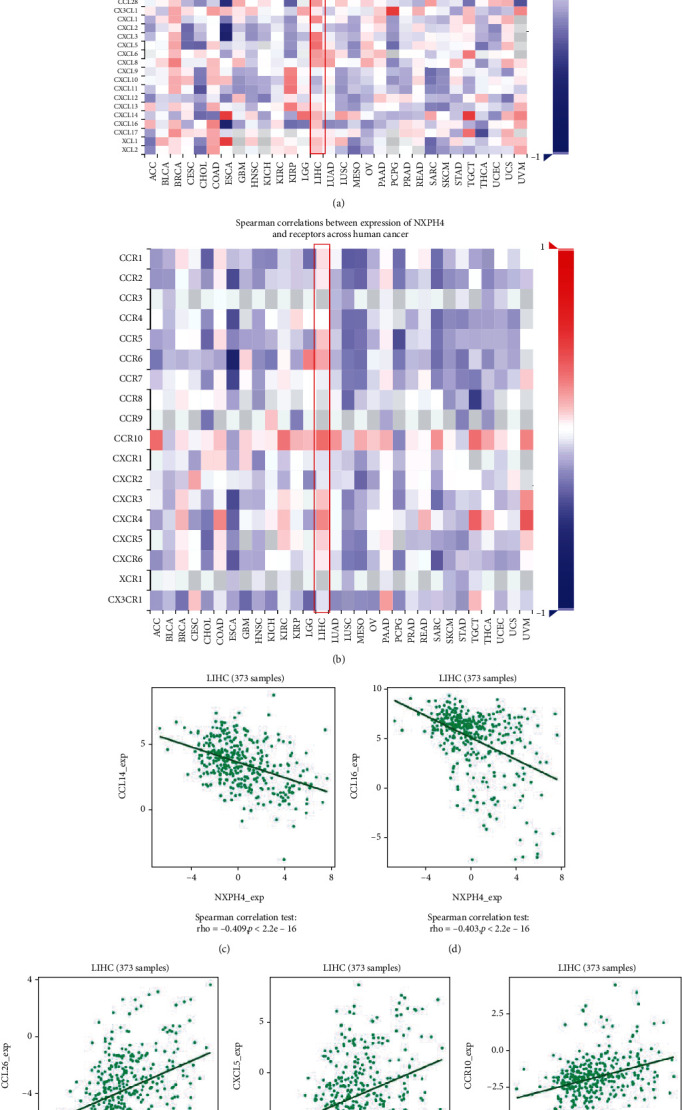
Correlation analysis between NXPH4 expression and chemokines and chemokines receptors. (a) Heat map analysis of the correlation between NXPH4 and chemokines in tumors. (b) Heat map analysis of the correlation between NXPH4 and receptors in tumors. (c–f) Chemokines: NXPH4 expression in HCC is correlated with CCL14, CCL16, CCL26, and CXCL15 (−0.3 < *r* < 0.3). (g) Chemokine receptors: NXPH4 expression in HCC is correlated with CCR10 (−0.3 < *r* < 0.3). Blue and red color stand for negative and positive correlations, separately.

Chemokines and chemokine receptors recruit immune cell into TME and affect tumor progression [21]. We estimated the relationship between the expression of NXPH4 and chemokines and chemokine receptors through TISIDB database. These heat map results displayed that NXPH4 expression was related with chemokines and chemokine receptors in cancers (Figures 8(a) and 8(b)). In HCC, NXPH4 expression was negatively correlated with CCL14 (Figure 8(c), rho = −0.409, *p* = 2.2*e* − 16) and CCL16 (Figure 8(d), rho = −0.403, *p* = 2.2*e* − 16). In addition, the NXPH4 expression was positively associated with CCL26 (Figure 8(e), rho = 0.36, *p* = 9.53*e* − 13), CXCL5 (Figure 8(f), rho = 0.31, *p* = 1.12*e* − 09), and CCR10 (Figure 8(g), rho = 0.324, *p* = 1.86*e* − 10). These results demonstrated that NXPH4 could play a significant role in tumor immunity.

### 3.6. Knocking Down the Expression of NXPH4 Inhibited Cell Proliferation, Migration, and Invasion

**Figure 9:**
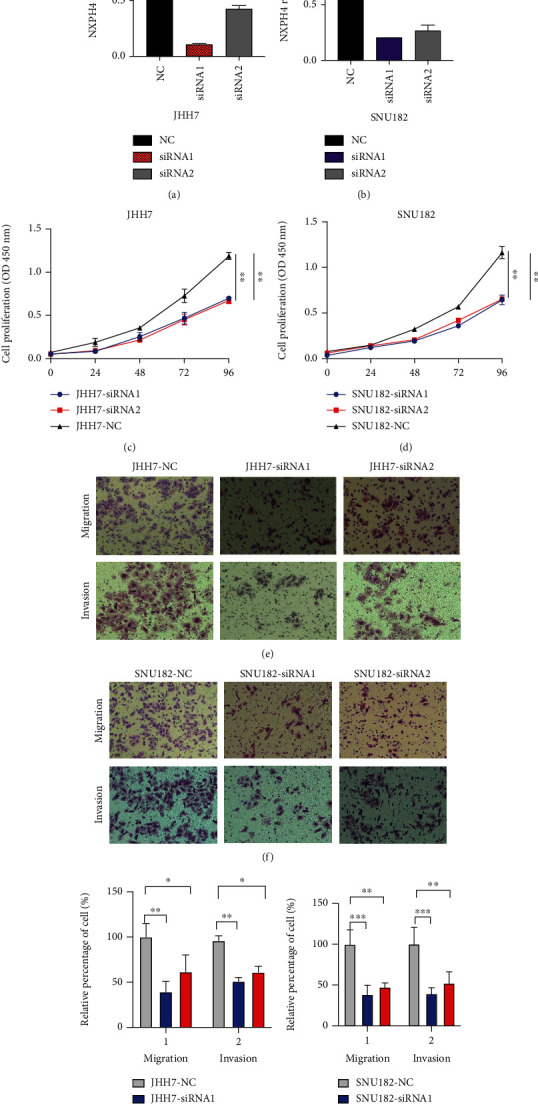
(a, b) NXPH4 mRNA was detected by quantitative RT-qPCR after 48 hours of transfection with NC siRNA or two different siRNAs targeting NXPH4 (siRNA1 and siRNA2). (c, d) CCK8 reagents were used to assay the proliferation ability at different time points after siRNA interference. (e, f) NXPH4 induces HCC cell (JHH7 and SNU182) migration and invasion. (g, h) The relative percentage of cells of NXPH4-induced migration and invasion in HCC cells (JHH7 and SNU182). Transwell assay to determine cell migration and invasion of JHH7 and SNU182 cells transfected with siRNA. ^∗^*p* < 0.05, ^∗∗^*p* < 0.01, and ^∗∗∗^*p* < 0.001.

We investigated the biological function of NXPH4 in JHH7 and SNU182 cells through knocking down the expression of NXPH4 via siRNA. The RT-qPCR results showed that siRNA1 and siRNA2 significantly decreased NXPH4 expression levels in JHH7 and SNU182 cells as compared to the NC group (Figures 9(a) and 9(b)). The cell proliferation assay results revealed that NXPH4 knockdown significantly suppressed JHH7 and SNU182 (Figures 9(c) and 9(d)) cell viability than the NC group. Moreover, we evaluated the impact of NXPH4 on migration and invasion of HCC cells. The Transwell invasion and migration assays showed that compared with the NC group, NXPH4 knockdown dramatically inhibited the number of invasive cells in JHH7 and SNU182 cells (Figures 9(e)–9(h)). These data demonstrated that NXPH4 has a prooncogenic role in HCC.

## 4. Discussion

Currently, only a few research has reported the function of NXPH4 in cancers. In the present study, we first conducted an integrated bioinformatics analysis to investigate the expression profiles, prognostic value, biological function, and potential regulatory pathways of NXPH4 in HCC. These bioinformatics analysis and basic research will lay a foundation for further comprehending the prognosis and treatment of patients with HCC. Based on bioinformatics analysis, we discovered that expression of NXPH4 was upregulated in HCC and related with T stage, N stage, clinical stage, grade, and status of TP53 mutation. Furthermore, we demonstrated that higher NXPH4 expression predicted worse survival time in HCC, and NXPH4 could act as an independent risk factor of HCC. These results illustrated that NXPH4 could be an effective biomarker of unfavorable prognosis to identify HCC with poor clinical outcomes.

For functional analyses, the construction of coexpression pattern identified the genes coexpressed with NXPH4. The results demonstrated that NXPH4 was positively related with the top coexpressed genes (PKM2, ENO2, SLC16A3, SAPCD2, and PNCK), and all these genes were associated with poor survival of HCC. PKM, ENO2, and SLC16A3 have been researched to have important effects on promoting tumor progression [22–26]. Based on the GO and KEGG pathway analyses, it was suggested that the NXPH4 coexpressed genes play important roles in metabolism and intracellular signaling transduction. Metabolism and immune escape are two basic characteristics of tumors. The metabolic environment could alter the immune response in the liver, enabling tumor cells to escape immunosurveillance.

Accumulating evidence shows that innate immune cells (such as macrophages, DC, NK cells, MDSC, and neutrophils) and adaptive immune cells (including B cells and T cells) can shape TME to promote tumor growth, metastasis, and treatment resistance [27, 28]. This crosstalk had been confirmed in HCC [29–32]. Based on the analyses of the expression NXPH4 with immune cell infiltration, immune cell gene markers, immune inhibitors, chemokines, and chemokine receptors, we found that the expression of NXPH4 was related with immune cell infiltration. Recently, a study on the effect of NXPH4 in the prognosis and immune cell infiltration of bladder cancer has been published [16]. We found that the infiltration levels of TIICs have significant difference in different NXPH4 expression level groups in HCC, including NK cells, regulatory T cells (Tregs), B cells naïve, resting mast cells, M1 macrophages, M2 macrophages, monocytes, resting dendritic cells, and M0 macrophages. However, in bladder cancer, there are only three types of TIICs, including memory B cells, resting dendritic cells, and M0 macrophages. Therefore, we speculated that NXPH4 could regulate tumor immune cell infiltration, but different tumors may have different types of immune cells. In TME, T lymphocytes have a critical function in cell-mediated immunity, attacking tumor cells with tumor specific antigens. Inhibitory immune checkpoints can inhibit T cell activation and promote T cell exhaustion [33]. Our study showed that upregulation of NXPH4 expression was positively related with several pivotal genes, such as PDCD1, CTLA4, LAG3, and TIM3. On the other hand, the expression of PDCD1, CTLA4, TIGIT, LAG3, and TIM3 was considerably higher in the high-NXPH4 expression group than in the low-NXPH4 expression group. The high expression of these genes in TME was associated to the weakening of T cell-mediated antitumoral immune responses [34]. Among these, PD1 (PDCD1) is the crucial regulator of effector T cell-mediated immune responses, which can lead to the exhaustion of T cell by exerting a suppressive signal to T cells with PD1 expression [35]. CTLA4 is a crucial inhibitor of T cell proliferation and promotes the immunosuppressive tumor microenvironment in HCC [20, 36]. Anti-PD1/PDL1 and anti-CTLA4 antibodies have been reported to be the major immunotherapy for several types of hematologic and solid malignancies. The measure of NXPH4 expression levels potentially contributed to provide new management for promoting efficient immunotherapy of HCC.

Chemokines and their receptors can regulate the directional migration of immune cells and directly or indirectly impact tumor cell proliferation, invasiveness, and metastasis [21]. The study displayed that the NXPH4 expression was negatively related with CCL14 and CCL16. A previous study showed that CCL14 played an essential role on the chemotaxis of T lymphocytes, monocytes, and eosinophils [37] and can act as a prognostic maker and tumor suppressor of HCC [38]. CCL16 binds to chemokine receptors (CCR1, CCR5, and CCR8) to activate angiogenesis in vascular endothelium and is related with prognosis in breast cancer and lung cancer [39, 40]. We speculated that the upregulated NXPH4 expression may suppress the migration of immune cells to tumor microenvironment, which may partially interpret how NXPH4 impact immune cell infiltration in HCC.

Finally, we found that knockdown of NXPH4 obviously inhibited cell proliferation, migration, and invasion ability in HCC cells. Based on bioinformatics analysis and experimental studies in vitro, we considered that NXPH4 could be a potential prognostic biomarker for HCC and closely associated with immune cell infiltration, cell proliferation, migration, and invasion of HCC. These findings should be confirmed in clinical studies and in vivo experiments.

## 5. Conclusion

In conclusion, the overexpression of NXPH4 was related with unfavorable prognosis and immune cell infiltration in HCC. Knockdown of NXPH4 inhibited proliferation, migration, and invasion ability of HCC cells. Our study contributes to better comprehend the role of NXPH4 in HCC and provide evidence for future studies.

## Data Availability

The data analyzed and utilized in this study are provided by the corresponding author upon reasonable request.

## Abbreviations

HCC: Hepatocellular carcinoma
NXPH4: Neurexophilin4
NSCLC: Non-small-cell lung cancer
TIICs: Tumor-infiltrating immune cells
Th1: T-helper1
Tfh: T cell follicular helper
DCs: Dendritic cells
NK cells: Natural killer cells
OS: Over survival
RFS: Recurrence free survival
PFS: Progression free survival
DSS: Disease specific survival
TAMs: Tumor-associated macrophages
MHC: Major histocompatibility complex
MDSC: Myeloid-derived suppressor cell
Treg: Regulatory T cell
PD-1: Programmed cell death protein 1
CTLA-4: Cytotoxic T lymphocyte antigen 4.

## Supplementary Materials

Supplementary Materials Figure S1: (A) boxplot shows the expression of NXPH4 in the TP53 mutation status group of HCC. (B) The boxplot showed the methylation level of NXPH4 in HCC (UALCAN). Figure S2: (A-D) functional enrichment analyses of the top 200 coexpressed genes of NXPH4 by GO and KEGG. Figure S3 (A-C): the infiltration of immune cells of top 3 genes correlated with NXPH4. Table S1: correlation analysis between NXPH4 and relate genes and markers of immune infiltration cells in HCC by TIMER.
