# Supplementary material for: Increased Expression of NXPH4 Correlates with Immune Cell Infiltration and Unfavorable Prognosis in Hepatocellular Carcinoma
Source: J Oncol. 2022 Oct 6;2022:5005747. doi: 10.1155/2022/5005747 (PMC9560829; doi:10.1155/2022/5005747)
Supplement: Supplementary Materials — Figure S1: (A) boxplot shows the expression of NXPH4 in the TP53 mutation status group of HCC. (B) The boxplot showed the methylation level of NXPH4 in HCC (UALCAN). Figure S2: (A-D) functional enrichment analyses of the top 200 coexpressed genes of NXPH4 by GO and KEGG. Figure S3 (A-C): the infiltration of immune cells of top 3 genes correlated with NXPH4. Table S1: correlation analysis between NXPH4 and relate genes and markers of immune infiltration cells in HCC by TIMER. [file 5005747.f1.zip › supplementary table1 (1).docx]

Supplementary: table1

| Table1 Correlation analysis between NXPH4 and relate genes and markers of immune infiltration cells in HCC by TIMER | | | | | |
| --- | --- | --- | --- | --- | --- |
| Description | gene markers | None cor | p-Value | purity cor | p-Value |
| CD8+T cell | CD8A | 0.156 | 0.00255 | 0.141 | 0.00872 |
|  | CD8B | 0.165 | 0.00139 | 0.151 | 0.00495 |
| CD4+T cell | CD4 | 0.213 | 3.14E-05 | 0.197 | 2.35E-04 |
|  | CD40LG(CD40L) | 0.126 | 0.0148 | 0.113 | 0.035 |
|  | CXCR4 | 0.362 | 6.07E-13 | 0.389 | 6.25E-14 |
| T cell(general) | CD3D | 0.322 | 2.04E-10 | 0.332 | 2.41E-10 |
|  | CD3E | 0.201 | 9.50E-05 | 0.203 | 1.52E-04 |
|  | CD2 | 0.208 | 5.26E-05 | 0.204 | 1.35E-04 |
| B cell | CD19 | 0.196 | 1.46E-04 | 0.187 | 4.79E-04 |
|  | CD79A | 0.164 | 1.49E-03 | 0.147 | 6.17E-03 |
| Monocyte | CD86 | 0.31 | 1.07E-09 | 0.336 | 1.49E-10 |
|  | CD115(CSF1R) | 0.243 | 2.25E-06 | 0.269 | 4.03E-07 |
| TAM | CCL2 | 0.171 | 9.67E-04 | 0.181 | 7.25E-04 |
|  | CD68 | 0.275 | 7.10E-08 | 0.287 | 5.52E-08 |
|  | IL10 | 0.223 | 1.42E-05 | 0.233 | 1.24E-05 |
| M1 macrophage | INOS(NOS2) | 0.01 | 0.85 | -0.009 | 0.87 |
|  | IRF5 | 0.215 | 3.02E-05 | 0.211 | 7.95E-05 |
|  | COX2(PTGS2) | 0.229 | 8.33E-06 | 0.252 | 2.22E-06 |
| M2 macrophage | CD163 | 0.071 | 0.175 | 0.067 | 0.212 |
|  | VSIG4 | 0.157 | 0.00247 | 0.173 | 0.00129 |
|  | MS4A4A | 0.135 | 0.00949 | 0.141 | 0.0088 |
| Neutrophils | CD66b(CEACAM8) | 0.047 | 0.369 | 0.057 | 0.289 |
|  | CD11b（ITGAM） | 0.336 | 3.27E-11 | 0.342 | 6.33E-11 |
|  | CCR7 | 0.055 | 0.29 | 0.026 | 0.635 |
| NK | KIR2DL1 | 0.004 | 0.938 | -0.003 | 0.948 |
|  | KIR2DL3 | 0.103 | 0.0469 | 0.082 | 0.129 |
|  | KIR2DL4 | 0.153 | 0.0032 | 0.138 | 0.0102 |
|  | KIR3DL1 | 0 | 0.994 | -0.019 | 0.728 |
|  | KIR3DL2 | 0.009 | 0.866 | -0.002 | 0.968 |
|  | KIR3DL3 | 0.032 | 0.543 | 0.027 | 0.618 |
|  | KIR3DS4 | -0.009 | 0.86 | -0.018 | 0.734 |
| Dendritic cell | HLA-DPB1 | 0.219 | 2.03E-05 | 0.224 | 2.59E-05 |
|  | HLA-DQB1 | 0.185 | 3.32E-04 | 0.192 | 3.43E-04 |
|  | HLA-DRA | 0.209 | 4.99E-05 | 0.215 | 5.90E-05 |
|  | HLA-DPA1 | 0.182 | 4.38E-04 | 0.187 | 4.97E-04 |
|  | BDCA-1(CD1C) | 0.049 | 0.343 | 0.038 | 0.481 |
|  | BDCA-4(NRP1) | 0.244 | 2.03E-06 | 0.246 | 3.80E-06 |
|  | CD11c(ITGAX) | 0.361 | 7.80E-13 | 0.373 | 8.31E-13 |
| Th1 | T-bet（TBX21) | 0.022 | 0.667 | -0.005 | 0.93 |
|  | STAT4 | 0.16 | 0.00201 | 0.14 | 0.0093 |
|  | STAT1 | 0.176 | 6.80E-04 | 0.162 | 0.00249 |
|  | INF-γ（INFG) | 0.182 | 4.42E-04 | 0.167 | 1.81E-03 |
|  | TNF-α（TNF) | 0.284 | 2.59E-08 | 0.285 | 7.45E-08 |
| Th2 | GATA3 | 0.264 | 2.45E-07 | 0.282 | 9.55E-08 |
|  | STAT6 | 0.032 | 0.544 | 0.026 | 0.63 |
|  | STAT5A | 0.217 | 2.40E-05 | 0.203 | 1.42E-04 |
|  | IL13 | 0.041 | 0.425 | 0.028 | 0.61 |
| Tfh | BCL6 | 0.057 | 0.272 | 0.072 | 0.184 |
|  | IL21 | -0.005 | 0.925 | -0.023 | 0.666 |
| Th17 | STAT3 | 0.215 | 3.08E-05 | 0.219 | 4.07E-05 |
|  | IL17A | -0.013 | 0.801 | -0.008 | 0.879 |
| Treg | FOXP3 | 0.029 | 0.572 | 0.024 | 0.658 |
|  | CCR8 | 0.241 | 2.57E-06 | 0.222 | 3.26E-05 |
|  | STAT5B | -0.033 | 0.532 | -0.028 | 0.607 |
|  | TGFβ(TGFB1) | 0.474 | 3.83E-22 | 0.494 | 1.13E-22 |
| T cell exhaustion | PD-1(PDCD1) | 0.294 | 7.47E-09 | 0.288 | 5.45E-08 |
|  | CTLA4 | 0.286 | 2.11E-08 | 0.285 | 7.57E-08 |
|  | LAG3 | 0.178 | 5.60E-04 | 0.155 | 3.82E-03 |
|  | TIM3(HAVCR2) | 0.3866 | 3.61E-13 | 0.4 | 1.08E-14 |
|  | GZMB | 0.044 | 4.00E-01 | 0.032 | 5.58E-01 |
